# Comparison of oral Dydrogesterone and 17-α hydroxyprogesterone caprate in the prevention of preterm birth

**DOI:** 10.1186/s12884-022-04509-1

**Published:** 2022-03-01

**Authors:** Fahimeh Alizadeh, Malihe Mahmoudinia, Masoumeh Mirteimoori, Lila pourali, Shabnam Niroumand

**Affiliations:** 1 Department of Obstetrics and Gynecology, Faculty of Medicines, Mashhad University of Medical Sciences, Mashhad, Iran; 2 Department of Obstetrics, Faculty of Medicines, Mashhad University of Medical Sciences, Mashhad, Iran; 3 Faculty of Medicines, Mashhad University of Medical Sciences, Mashhad, Iran

**Keywords:** 17α-Hydroxyprogesterone caproate, Preterm labor, Preterm birth, Dydrogesterone

## Abstract

**Background:**

Preterm birth (PTB) remains a significant problem in obstetric care. Progesterone supplements are believed to reduce the rate of preterm labor, but formulation, type of administration, and dosage varies in different studies. This study was performed to compare oral Dydrogesterone with intramuscular 17α-hydroxyprogesterone caproate (17α-OHPC) administration in prevention of PTB.

**Methods:**

In this randomized clinical trial, we studied 150 women with singleton pregnancy in 28^Th^-34^Th^ Gestational week, who had received tocolytic treatment for preterm labor. Participants were divided to receive 30 mg oral Dydrogesterone daily, 250 mg intramuscular 17α-OHPC weekly, or no intervention (control group). All treatments were continued until 37^Th^ Week or delivery, whichever occurred earlier. Obstetric outcomes, including latency period, gestational age at delivery, birth weight, neonatal intensive care unit (NICU) admission, and neonatal mortality were recorded. All patients were monitored biweekly until delivery.

**Results:**

Baseline gestational age was not significantly different between groups. Latency period was significantly longer in the progesterone group compared with Dydrogesterone and control groups (41.06 ± 17.29 vs. 29.44 ± 15.6 and 22.20 ± 4.51 days, respectively; *P* < 0.001). The progesterone group showed significantly better results compared with the other two groups, in terms of gestational age at delivery, birth weight, and Apgar score (*P* < 0.001). None of the participants showed severe complications, stillbirth, or gestational diabetes.

**Conclusion:**

Progesterone caproate can strongly prolong the latency period and improve neonatal outcomes and therefore, is superior to oral Dydrogesterone in the prevention of PTB.

## Background

Preterm birth (PTB) has long been a critical challenge and social concern for healthcare providers. It can lead to prematurity and put infants at a higher risk of developing various morbidities including intraventricular hemorrhage, necrotizing enterocolitis, respiratory distress syndrome, sepsis, severe neurological deficits, and even mortality. Therefore, the search for optimal preventive options against PTB is imperative [1–3].

For decades, it has been the mission of physicians and scientists to find preventive agents against PTB, especially in women with the risk factors, including history of PTB, short cervical length, multifetal pregnancy, infectious diseases, genetic predisposition, smoking, advanced maternal age, uterine anomaly, and history of curettage or cervical conization [4–7].

There are different routes of administration and progesterone types, which have been studied previously [8–11]. 17-α-hydroxyprogesterone caproate (17α-OHPC) is a synthetic derivative of 17 hydroxyprogesterone (17-OHP), which is inactivated when orally administered, thus it is injected intramuscularly [12].

Dydrogesterone (6-dehydro-9b, 10a-progesterone) is a potent, orally administered progestogen, similar to endogenous progesterone in its molecular structure and pharmacological effects, with a high affinity for the progesterone receptor [13]. The preferred administration route of Dydrogesterone makes it an ideal alternative if it proves to be adequately efficient in preventing PTB.

Previous studies suggest that progesterone agents may not perform similarly in different pathological processes leading to PTB. While 17α-OHPC has been proven to cause the least amount of side effects, a recent study indicated that it does not delay the delivery or reduce the rate of PTB in women with a short cervix [14].

To date, the most sought prevention methods for PTB in women with high risk of PTB is progesterone supplement therapy. Progesterone has been proven to delay the delivery in order for the pregnancy to reach its physiologic term. There have been numerous studies conducted on supplementation of progesterone for PTB prevention [11, 12, 15, 16]. The primary objective of this study was to investigate the efficacy of oral Dydrogesterone and 17α-OHPC in preventing PTB in pregnant women with preterm labor.

## Methods

### Study setting and ethics

This open-label randomized controlled trial was carried out on pregnant women with preterm labor who referred to the obstetric units of Imam Reza, Ghaem, and Um-al-banin hospitals in Mashhad, Iran, during 2018–2019.

All subjects were informed and consented before their enrollment. The ethics committee of Mashhad University of Medical Sciences approved the study with the approval code of IR.MUMS.medical.REC.1397.378. We had also registered the study in the Iranian registry of clinical trials (registered number: IRCT20181207041879N1) in 24/02/2019.

### Patients

Keeping an alpha error of 0.05 and a beta equal to 0.2, the sample size was calculated to be 150 (50 subjects in each group), using the samps function of state version 11 (Stata Corporation, TX, USA). However, the sample size was extended to 165, assuming a possible 10% dropout rate in this population. Finally, 165 pregnant women with preterm labor who were admitted to our centers during the study period and met our inclusion criteria were recruited through convenience non-random sampling method.

Non-smoker women aged 18–35 years with singleton pregnancies in 28^Th^-34^Th^ Weeks of gestation (based on the first trimester ultrasound) who underwent magnesium sulfate treatment for preterm were included in the study if they had no contraction after 12 h and no recurrence of preterm labor pain after 48 h. The patients received two 12-mg doses of intravenous betamethasone within 48 h, before they enter the study.

Exclusion criteria were as follows: placenta previa, premature rupture of membranes, chorioamnionitis, severe pre-eclampsia, dilatation > 4 cm, fetal anomalies, cervical cerclage, prior progesterone use, contraindications to receiving progesterone or tocolytic agents, and voluntary withdrawal. Participants with irregular drug use were also excluded from the study.

### Data collection

Demographic data, including maternal age and gestational age upon admission were gathered in checklists. Obstetric outcomes, including gestational age at the time of delivery, latency period, mode of delivery, birth weight (and percentile), Apgar score, stillbirth, and NICU admission was also collected in the checklists.

The research team visited the participants every two weeks during the study to assess the recurrence of preterm labor pain, check the proper and regular use of medications, and investigate gestational diabetes. Moreover, subjects were monitored for any side effects of the treatment, including nausea, fatigue, pruritus, bruises, edema, pain at the site of injection, and progesterone-induced thromboembolism during the visits and the complications were recorded.

### Intervention

The participants were randomly assigned to three different groups of progesterone (Group 1) (*N* = 55), Dydrogesterone (Group 2) (*N* = 55), and control (Group 3) (*N* = 55) after signing informed consent forms. Random allocation was done using a computer-generated list of random numbers.

The Dydrogesterone group received Dydrogesterone 10-mg tablets (Aburaihan Pharmaceutical Co., Tehran, Iran) three times daily, following meals. The subjects in the progesterone group underwent treatment with weekly intramuscular injections (250 mg) of 17α-OHPC (Femolife™, Aburaihan Pharmaceutical Co., Tehran, Iran). The control group received no intervention.

### Statistical analyses

We used SPSS software (version 24 for Windows; IBM Statistics, USA) to carry out statistical analyses. Kolmogorov–Smirnov test was used to assess normal distribution of data. One-way ANOVA test, Kruskal–Wallis test, Chi-square test, and Fisher exact test were used to compare data between groups of participants. Multiple stepwise linear regression was performed to assess the independent effect of variables on latency period and also binary logistic regression was done for variables effect on NICU admission. A *P* < 0.05 was considered statistically significant in all tests, except for Kolmogorov–Smirnov test in which *P* < 0.01 was considered significant.

## Results

**Fig. 1:**
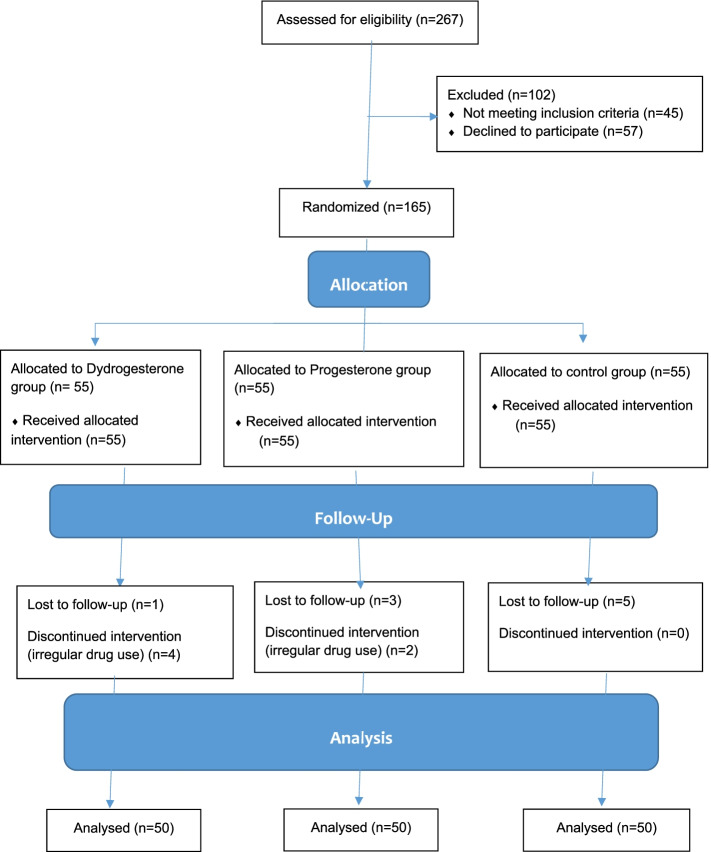
CONSORT flow diagram of the study

Overall, 165 pregnant women in three groups of Dydrogesterone (*N* = 55), progesterone (*N* = 55), and control (*N* = 55) were enrolled initially in the study. The intervention was discontinued in six participants (4 in the Dydrogesterone group and 2 in the progesterone group) due to irregular drug use. Besides, nine participants (5 in the control group, 3 in the progesterone group, and 1 in the Dydrogesterone group) were lost to follow-up due to poor compliance and inaccessibility. Eventually, 150 participants completed the treatment and entered our analysis (Fig. 1).

Table 1 compares baseline characteristics between the three groups. As it implies, the two groups showed no significant difference in baseline gestational age (*P* = 0.707). However, they had a significant difference regarding the maternal age (*P* = 0.017).

**Table 1:** Baseline characteristics of participants

Baseline Variable	Group 1 (*N* = 50)	Group 2 (*N* = 50)	Group 3 (*N* = 50)	*P*
Maternal age (years)	28.60 ± 4.04	26.53 ± 4.55	28.71 ± 3.98	0.017^a^
GA on admission (days)	220.50 ± 13.30	219.88 ± 13.81	222.04 ± 12.84	0.707^a^
GA on admission (weeks)	31.50 ± 1.90	31.41 ± 1.97	31.72 ± 1.83	0.69^a^
GA on admission categories (N (%))				
28 ≤ GA < 30	10 (20%)	16 (32%)	11 (22%)	0.55^b^
30 ≤ GA < 32	12 (24%)	8 (16%)	13 (26%)	
32 ≤ GA < 35	28 (56%)	26 (52%)	26 (52%)	

*GA* Gestational age

^a^One-way ANOVA test

^b^Chi-square test

The progesterone group showed a significantly longer latency period (41.06 ± 17.29 days) compared with the Dydrogesterone (29.44 ± 15.65 days) and control group (22.20 ± 4.51 days) (*P* < 0.001). There were also significant differences between the study groups in terms of gestational age at delivery, birth weight, and Apgar score (*P* < 0.001). However, no significant difference was observed between the groups in the mode of delivery (*P* = 0.182) and rate of NICU admissions (*P* = 0.050). None of the pregnancies ended in stillbirth. Main outcomes are compared between the three study groups in Table 2.

**Table 2:** Comparison of main outcomes between the study groups

Outcome		Group 1 (*N* = 50)	Group 2 (*N* = 50)	Group 3 (*N* = 50)	*P*
**GA at delivery (days)**		261.56 ± 14.90	249.32 ± 17.23	244.24 ± 18.08	< 0.001^a^
**GA at delivery (weeks)**		37.36 ± 2.12	35.61 ± 2.46	34.88 ± 2.59	< 0.001^a^
**Latency period (days)**		41.06 ± 17.29	29.44 ± 15.65	22.20 ± 14.51	< 0.001^a^
**Latency period (mean difference)**		Group1&2	Group2&3	Group1&3	
		11.62 ± 3.29	7.32 ± 3.02	18.94 ± 3.20	
**Birth weight (2)**		3042 ± 678	2424 ± 720	2341 ± 707	< 0.001^a^
**Weight percentile**	< 5%	3 (6%)	3 (6%)	4 (8%)	< 0.001^b^
	5–10%	0 (0%)	11 (22%)	12 (24%)	
	10–50%	22 (44%)	27 (54%)	24 (48%)	
	50–90%	23 (46%)	9 (18%)	10 (20%)	
	> 90%	2 (4%)	0 (0%)	0 (0%)	
**Apgar score**		10 (10–10)	9.5 (8–10)	8 (7–10)	< 0.001^b^
**NICU admissions**		11 (22%)	20 (40%)	22 (44%)	0.050^c^
**Mode of delivery**	ND	26 (52%)	30 (60%)	15 (30%)	0.182^c^
	CS	24 (48%)	20 (40%)	35 (70%)	
**GA at delivery (weeks cat)**	> 37 weeks	35 (70%)	18 (36%)	11 (22%)	< 0.001
	< 37 weeks	15 (30%)	32 (64%)	39 (78%)	

*GA* Gestational age, *NICU* Neonatal intensive care unit, *ND* Natural delivery, *CS* Cesarean section

^a^One-way ANOVA test

^b^Kruskal-Wallis test

^c^Chi-square test

**Fig. 2:**
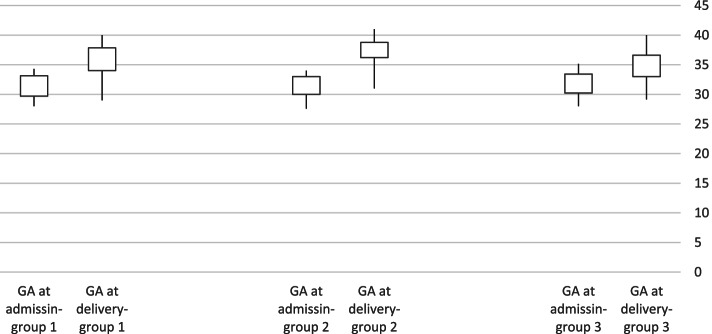
GA at admission and delivery in each study groups

Between group analyses, using Post-Hoc Tukey test, showed that there were no significant differences between the Dydrogesterone and the control group regarding gestational age at delivery (*P* = 0.279), latency period (*P* = 0.059), birth weight (*P* = 0.958), and Apgar score (*P* = 0.242). Moreover, no significant differences were found between the Dydrogesterone and the control group in terms of mode of delivery (*P* = 0.295), rate of NICU admissions (*P* = 0.685), and birth weight percentiles (*P* = 0.840). GA at admission and delivery in each group were shown as a box-whisker plot in Fig. 2.

Multiple stepwise linear regression was done to assess the independent effect of other variables on latency periods. As Table 3 showed, first pregnancy age and also birth weight had statistically significant independent effect on latency period (both *P* < 0.001). Also, binary logistic regression for variables effect on NICU admission was performed and according to Table 3, first pregnancy age (*P* = 0.03) and end pregnancy age (*P* < 0.001) had independent effect on the rate of NICU admission.

**Table 3:** Regression on variables predicting the latency period and NICU admission

Dependent variable	Independent variables	OR	95% C.I		*p*-value
Latency period (days)^a^	First pregnancy age	-0.46	-.065	-.027	< 0.001
	Mother age	-0.094	-.056	0.37	0.69
	Birth weight	0.004	0.003	0.006	< 0.001
NICU admission^b^	First pregnancy age	0.96	0.93	0.99	0.03
	End pregnancy age	0.92	0.89	0.95	< 0.001
	Mother age	1.04	0.96	1.13	0.29
	Kind of delivery (CS)	1.27	0.50	3.21	0.60

^a^Based on Multiple stepwise linear regression

^b^Based on Binary logistic regression

Considering the side effects of treatment, nausea was seen in only five participants in the Dydrogesterone group (10%) and three women in the progesterone group (6%) reported bruises at the injection site, which did not reoccur in the following weeks of treatment. None of the subjects experienced severe complications such as thromboembolism. Furthermore, none of the participants developed gestational diabetes.

## Discussion

Prevention is the most effective treatment for PTB. Currently, the widely used method for prevention of PTB is adjuvant progesterone therapy. The use of progesterone has also been recommended by the American Congress of obstetricians and gynecologists (ACOG) to prevent PTB in pregnant women with risk factors such as preterm labor [12].

There has been a growing advancement in the understanding of progesterone signaling pathways lately, which has shed light into novel targets for progestin-based therapies to prevent PTB. It has been suggested that new selective progestin agents can boost anti-inflammatory activities that prevent preterm labor [17].

Herein, we studied the therapeutic effect of 17α-OHPC and Dydrogesterone in comparison with no intervention at all for the prevention of PTB and delaying the delivery. We observed no significant differences in outcomes between the Dydrogesterone and control groups.

However, in most study participants, with careful care and surveillance in accordance with protocol, we could prolong gestational age until 34 weeks and most of the infants were mature but nevertheless we found that 17α-OHPC induced a markedly longer latency period and subsequently led to more favorable obstetric and neonatal outcomes, compared to both the Dydrogesterone and the control groups. Moreover, we observed no serious side effects in the participants and only few cases of nausea and bruises at the injection site occurred, which were trivial and resolved spontaneously without any specific treatment.

Shahgheibi and colleagues compared weekly intramuscular injections of 17α-OHPC (250 mg) with placebo in 100 high-risk pregnant women in 24^Th^-34^Th^ Weeks of gestation. Consistent with our findings, they reported a significant effect for 17α-OHPC injection on prevention of PTB [18]. This consistency is important because their study had similar methodology and sample size and this can somehow confirm the reliability of our results. Similar results have also been reported by Ibrahim et al. In a randomized trial comparing weekly intramuscular injections of 17α-OHPC with placebo in prevention of PTB among 50 women with a prior history of PTB [19].

A recent open-label clinical trial by Pustotina compared the efficacy of 17-OH progesterone, Dydrogesterone, and vaginal or oral micronized progesterone with cerclage to prevent PTB in women with a short cervix. They studied 95 women with singleton pregnancy at 15–24 weeks and short cervix, of whom 60 had symptoms of preterm labor. They divided the patients to receive 30 mg daily oral Dydrogesterone, 250 mg weekly 17OHP, 400 mg daily oral progesterone, or 400 mg daily vaginal progesterone capsules [20].

In contrast with our findings, Pustotina found that vaginal progesterone prevents about 94% of the PTBs in pregnant women with a short cervix, while Dydrogesterone, 17-OH progesterone, and oral micronized progesterone were associated with PTB in 91.7% of cases. She also found that vaginal progesterone is associated with lower risk of low birth weight [20]. The inconsistency between our results and that of the mentioned study probably stems from the fundamental difference in our sample population and methods used. Our sample size was relatively larger and our participants were all in 28^Th^-34^Th^ Gestational weeks and had evident preterm labor.

Fonseca et al. Also showed the efficacy of vaginal progesterone at a dose of 200 mg daily in the prevention of PTB in women with a short cervix. Nevertheless, they did not find any significant reduction in neonatal morbidity with this intervention [21]. This finding is in line with the results of the prior mentioned study by pustotina, which indicated that vaginal progesterone has a dose-dependent effect on pregnancy outcomes [20].

Born and colleagues randomized 70 pregnant women with preterm labor to receive either 400 mg daily vaginal progesterone suppository or no treatment. They found that vaginal progesterone not only significantly increases the latency period, but also decreases the neonatal complications such as low birth weight and respiratory distress syndrome. However, it was not found to be effective in reducing the rate of NICU admissions and sepsis [22]. These results are not directly comparable with our findings because of the different route of administration and type of progesterone, but somehow support the main theoretical basis of our findings, i.e. The beneficial effects of progesterone in preventing PTB and neonatal complications.

We did not compare Dydrogesterone and 17α-OHPC with vaginal or oral micronized progesterone. However, a previous study compared daily vaginal micronized progesterone (200 mg) and weekly intramuscular 17α-OHPC (250 mg) with no progesterone, in 60 women at 20–24 weeks gestation and reported equal efficacy for both intramuscular and vaginal progesterone in the prevention of preterm labor [23].

Norman et al. in a large-scale study compared 200 mg daily vaginal progesterone and placebo in prevention of PTB and adverse neonatal outcomes. In contrast with the results of the above mentioned studies, they reported that vaginal progesterone was not associated with reduced risk of PTB or neonatal complications [24].

A randomized, double blinded, placebo controlled trial by Areeruk and Phupong investigated the efficacy of oral Dydrogesterone (20 mg daily) in the management of preterm labor in 48 pregnant women at 24^Th^-34^Th^ Gestational weeks. They found no significant difference between Dydrogesterone and placebo groups in terms of recurrent uterine contractions, latency period, gestational age at delivery, mode of delivery, birth weight, Apgar score, neonatal morbidity, and mortality [25]. These results were completely compatible with our results regarding the comparison of outcomes between Dydrogesterone group and the controls, even though we used higher doses of Dydrogesterone compared with that in Areeruk’s study.

To the best of our knowledge, few studies with comparable sample size have investigated the efficacy of both 17α-OHPC and Dydrogesterone in a controlled trial design to prevent PTB in high-risk pregnancies. However, there are several limitations to our study. First, our study was not blinded and this could put the findings at risk of possible bias. Second, although we tried to minimize the baseline differences between the study groups, we could not completely match the maternal age of participants. This might have had a confounding effect on our findings. Lastly, including vaginal progesterone along with our two interventions might have led to a better understanding of the effect of different routes of administration on the preventive role of progesterone in the PTB.17α-OHPC injection can strongly prolong the latency period and improve neonatal outcomes and therefore, is superior to oral Dydrogesterone in the prevention of PTB.

In conclusion, although 17α-OHPC may be a more invasive option in the prevention of PTB, its benefits outweigh its drawbacks and it can be recommended for women with preterm labor and risk of PTB. Further studies are required to improve diagnostic and therapeutic strategies for prevention of PTB, especially in women with high-risk pregnancies.

## Data Availability

The datasets generated for this study are available on request to the corresponding author.
